# Dynamic model of respiratory infectious disease transmission by population mobility based on city network

**DOI:** 10.1098/rsos.221232

**Published:** 2022-11-30

**Authors:** Zuiyuan Guo, Jiangfan Li, Guangquan Xiao, Lili Gong, Yayu Wang

**Affiliations:** ^1^ Infectious Disease Prevention and Control Division, Beibu Zhanqu Center for Disease Control and Prevention, Shenyang, People's Republic of China; ^2^ Department of Psychiatry, General Hospital of Northern Theater Command, Shenyang, People's Republic of China

**Keywords:** city network, population mobility, respiratory infectious disease, dynamic model

## Abstract

In modern societies, newly emerging infectious diseases spread rapidly between regions owing to frequent contact between people, causing considerable social and economic impacts. In this study, first, a scale-free city network was established, and then the shortest path between any two nodes was determined. Second, the movement path of tourists was designed based on the shortest path. Subsequently, every infected person's information, such as the city, infection time, onset and hospitalization, was confirmed based on their movement path. Third, the features of the transmission path and time distribution of the epidemic were characterized after summarizing the information. Finally, the reliability of the model was verified. The number of citizens and tourists in every city remained stable during this time. The results indicated that a larger basic reproduction number (*R*_0_) and population outflow rate signify a faster growth rate of infected people in each city in the network. Compared with small and medium-sized cities, the epidemic spread faster in central cities. Population mobility was the decisive factor causing the spread of the epidemic to other areas. Therefore, the rapid spread of epidemics can be prevented by swiftly reducing the flow of people between cities.

## 1. Introduction

Infectious respiratory diseases are transmitted through the mobility of people, which spreads an epidemic from one city to a whole nation or even worldwide. For example, the H1N1 influenza, which originated in the United States and Mexico in April 2009, spread to 40 countries in a month [1]. In addition, the Omicron variant of SARS-CoV-2 rapidly became the primary epidemic strain after it was first discovered in mainland China in December 2021. Subsequently, it spread to 28 of the 32 provinces in the mainland in less than six months [2,3]. Modern transportation facilitates people's travel, and in turn, the transmission of epidemics. For example, in China, the dense high-speed rail network that carries hundreds of millions of travelling people spread the COVID-19 epidemic, which first appeared in Wuhan in 2019, to several provinces and cities of the country through the transportation of people during the Spring Festival. Because population mobility is crucial in transmitting an epidemic, the dynamic epidemic transmission process through population mobility between different regions and quantitative analysis of the epidemic trend should be studied. Such studies can provide a clear understanding of the transmission mechanism of the epidemic to formulate targeted prevention and control measures.

Cities that are connected through traffic constitute a complex network. In this network, nodes and edges represent cities and traffic routes, respectively. However, obtaining real data regarding population mobility is challenging because people can choose to travel by road, railway or civil aviation. Therefore, a virtual city network is generally established to facilitate the research. The distribution degree of city networks is uneven because there are central cities with large populations and well-developed transportation, and there are small cities with small populations and inconvenient transportation facilities. Nevertheless, some transportation networks among cities are scale-free [4,5]. Therefore, the city network is assumed to be a scale-free network, implying that some nodes have many connections while most of the nodes have fewer connections. Moreover, the degree of the network conforms to the power-law distribution. The stronger the hub role of a city, the larger the number of people and the higher the degree of the nodes.

A dynamic model should be developed to study the transmission of infectious diseases in the city networks. Therefore, numerous models of infectious diseases have been established [6–12] that play an essential role in comprehending the transmission mechanism of infectious diseases on complicated networks. However, these models are multi-group models, i.e. macro models; hence, they cannot describe the travel process at the individual level. In addition, some models do not follow the research methods of infectious disease dynamics, although the spread of the epidemic in the city networks has been established [8]. Few dynamic models have been used to simulate and analyse the dynamic characteristics of epidemic transmission by population mobility in a city network.

The individual-based dynamic model can simulate the movement paths of individuals and their complete infection process including onset, hospitalization and rehabilitation, from a micro perspective. Moreover, it can aggregate individuals into groups to quantitatively characterize the epidemiological features of the epidemic from a macro perspective. We have previously used the individual-based dynamic model to study the transmission dynamics of various infectious diseases. Although replacing differential equations with computer programming was a common point in the studies conducted, the research subject and technical route of each model differed owing to the different types of crowd activities studied. This study analysed the spread of infectious diseases through the flow of people in an urban network. We first established a scale-free network, then searched for the shortest distance between any two nodes, and finally designed a travel plan for the population. On this basis, we simulated the transmission process of Omicron. The implementation steps were as follows: first, we updated the local and foreign populations of each city in the network daily. Then, we simulated the dynamic changes in population mobility and epidemic transmission by combining them, adjusted the population mobility scale according to the patient's hospital stay, and finally counted the time distribution of epidemic transmission in each city. Consequently, we found that the original intention, basic architecture, program design and discipline of this model differ from those of previous studies.

## 2. Methods

### 2.1. Data source

Data such as the incubation period, symptom period, hospitalization rate and hospitalization period of Omicron infection were obtained from the published literature. The network and population parameters were assumed on the basis of objective facts. The parameters are listed in table 1.

**Table 1.** Model parameters.

description	distribution characteristics	numerical values	sources
incubation period	lognormal distribution	$\begin{array}{rl} & \mu =5.2\\ & \sigma =0.87 \end{array}$	[13]
proportion of patients requiring hospitalization to the total number of patients	constants	0.02	[14]
duration of symptoms of the patients with mild symptoms	constant	2–7 d	[14]
delay between symptom onset and hospitalization	constant	4–12 d	[14]
length of stay	constant	mean = 4.0; s.d. = 3.7	[15]
population outflow rate	constants	0.01	assumed

### 2.2. Model establishment

#### 2.2.1. Prerequisite

(i) A certain proportion of citizens commute from their resident city to several other cities and return daily; the route of each traveller is a loop. (ii) Tourists make a travel plan before the travel, including travel destinations and stay duration at each destination. (iii) The shortest travel time between the two cities is the standard for selecting the best route. Tourists do not enter the cities they pass through, and the number of destinations per trip conforms to the Poisson distribution with a mean value of 1. (iv) Each traveller's travel time between any two cities in the network is fixed. (v) The first case emerges in City 1, and all citizens are susceptible at the initial stage of the epidemic; the number of second-generation infected persons of each infector conforms to the Poisson distribution with a mean value of *R*_0_. (vi) Everyone will experience an incubation period after infection. Mild patients will recover after experiencing a symptomatic period (infectious period) without hospitalization. By contrast, severe patients will be hospitalized after the symptomatic period (infectious period). These patients will not infect other patients during hospitalization because of protective measures implemented by the medical staff; and patients recover after the treatment. Mild and severe patients will acquire immunity after recovery. (vii) The travel plan of mild patients at the time of onset and severe patients before hospitalization will not be affected. The severe patients will be hospitalized in the city which they are visiting or residing in during infection, and they will continue to travel according to the original travel plan after being discharged. (viii) The local citizens and foreign tourists in the city are evenly mixed and randomly chosen as the next generation of infected people based on their population ratio.

#### 2.2.2. Total framework

**Figure 1.**
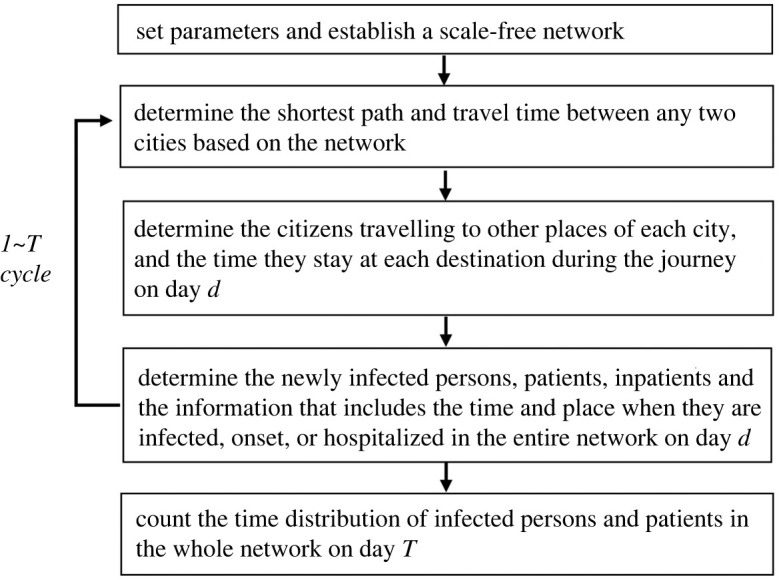
Framework of the proposed model.

First, a scale-free city network was established. Then, the population of each city and the travel time between any two cities were set, and the path between any two cities with the shortest travel time was determined. Thereafter, the travel time of each shortest route and all contained cities were saved in a data frame. Subsequently, the time cycle was started from the first day, and incremented one day per cycle. At the beginning of cycle *d*, all tourists who ended their trip on day *d* − 1 were returned to their origins. Then, the local tourists who started their trip on day *d,* from each city were randomly selected based on the existing local citizens and the population outflow rate (i.e. the proportion of the number of local citizens who travel to other cities every day to the total number of local citizens in the city). The travel plans, i.e. the time of arrival and departure from each city of all tourists in the network on day *d,* were saved in a data frame denoted *Outlanders*. Based on this data frame, the city where the patients are infected, sick and hospitalized can be determined. Thereafter, the epidemic spread according to the rule that each infected person causes new infected persons on an average of *R*_0_, and the information, such as city, personal ID, time and city of infection and onset, of all newly infected persons, patients and inpatients on the same day were saved in another data frame *D*. The time distribution of infected persons, patients and inpatients in each city was statistically analysed after the time cycle ended based on *D*. The design framework and the global algorithm of the model are shown in figure 1 and electronic supplementary material, figure S1, respectively.

#### 2.2.3. City network establishment

The city network was established using the algorithm of the scale-free network model proposed by Barabási & Albert [16]. Starting from three discrete nodes, a new node was added each time when connecting it with three existing nodes. The probability of a new node connecting with an existing node was proportional to the degree of the node. Notably, based on the growth and preferential selection strategy, the scale of the network can eventually be made stable; in other words, the degree distribution of the network will not vary with time. The nodes represented cities and the edges represented traffic routes. The weight of each edge connecting the two cities indicated the driving time, which was randomly selected in the 1–5 h range.

#### 2.2.4. Finding the shortest path

The shortest route was defined as the path with the shortest driving time between two cities. Various algorithms could be used to find the shortest path between two nodes in a network. In particular, the Dijkstra algorithm was selected, which could find the shortest path from a single source point to other nodes in a directed graph. Furthermore, the path that took the shortest time between two points could be obtained by inputting a weight-directed graph [17]. The basic operation of Dijkstra algorithm is edge expansion: when we search for the shortest path from point *v*_s_ to *v*_m_, if there exists an edge from point *v_i_* to *v*_m_, the shortest path from *v*_s_ to *v*_m_ can be expanded by adding the edge *e_i_*_m_ to the tail. The length of this path is *d* [*i*] + *w_i_*_m_. If this value is smaller than the current known value of *d* [m], replace the current value of *d* [m] with the new value. The operation continues expanding edges until *d* [m] represents the cost of the shortest path from *v*_s_ to *v*_m_. After the algorithm is completed, we store the shortest route and travel time between any two cities in the data frame *Route*. For the specifics regarding the implemented algorithm, see the electronic supplementary material, appendix, lines 76–143.

#### 2.2.5. Population mobility simulation

The number of foreign tourists and local citizens as well as their IDs in each city of the network must be stored and counted every day owing to the mobility of the daily population. The specific steps employed to perform this count were as follows: on day *d*, (i) count the tourists (local citizens) in each city who returned to the origin on day *d* − 1. (ii) Randomly select the tourists who went out on day *d* based on the existing number of local citizens (as patients do not travel during hospitalization, such patients were removed from local citizens) in the city and the population outflow rate. (iii) Add their travel plans to the *Outlanders* data frame. The information in each row of the data frame includes the traveller's origin, personal ID and the time of arrival and departure at each destination. (iv) Delete the corresponding rows of tourists who returned to the origins on day *d −* 1 in *Outlanders* after tourists in all cities were added, so that the data frame always contains the information of all tourists on day *d*. The city where each traveller was at any time could be determined from the data frame. For the specifics regarding the implemented algorithm, see the electronic supplementary material, appendix, lines 193–334.

#### 2.2.6. Transmission of diseases

The time distribution of infected persons and patients in each city should be counted. Therefore, it is necessary to determine the city where they were infected and attacked. For this, a four-row data frame named *State* was established, and each column was used to save relevant information of each newly infected person, patient, inpatient or discharged person (identified with numbers 1 to 4, respectively), including the origin, personal ID and infection time (or onset, hospitalization, discharge). The information of persons on day *d*, referred to as *State0*, was determined according to the time information in *State*. Moreover, the city where each person was at the time of infection, onset and hospitalization (the city was determined according to *Outlanders* if a person was exiting the city) was further determined based on the information of each column of *State0* and *Outlanders* on day *d*. If a column corresponded to the infected person's information, such as the origin, personal ID, infected time, infected city, the number of second-generation infected persons and their infected time, it was added to the last row in data frame *D*, which was used to save the information of all infected persons, and each row represented an infected person. If a column of *State0* corresponded to the information of a patient, inpatient or discharged person, the city where the person was determined to be onset, hospitalized, or discharged, it was added into *D*. After the above operations are completed, *State0* must be deleted from *State*. For the specifics regarding the implemented algorithm, see the electronic supplementary material, appendix, lines 336–484.

When the time cycle is over, we obtain *D* that fully records the information of all infected persons from day 1 to day *d*. *D* describes the time distribution characteristics of the epidemic situation and the entire process of infection spreading in the network.

### 2.3. Sensitivity analyses

Because the actual statistical data could not be used to fit data to estimate the parameters and verify the reliability of the model, sensitivity analyses of the model were performed in terms of two important parameters of the model—*R*_0_ and population outflow rate. Partial rank correlation coefficients (PRCC) and Latin hypercube sampling (LHS) were used to conduct the sensitivity analyses. PRCC-LHS is an efficient and reliable sampling-based sensitivity analysis method providing a measure of monotonicity between a set of parameters and the model output after removing the linear effects of all parameters, except the parameter of interest [18]. Each parameter interval was divided into N smaller and equal intervals, and one sample was selected randomly from each interval [18,19]. A standard coefficient denoting the correlation between the parameter and the model output was calculated. All analyses were conducted using Matlab R2019a software (MathWorks, Natick, MA, USA).

## 3. Results

### 3.1. City network

**Figure 2.**
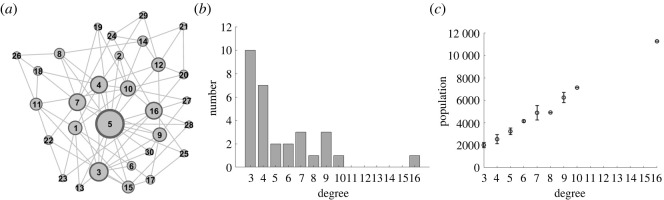
Topological structure and static characteristics of urban network. (*a*) The city network plan. Nodes represent cities and edges represent traffic routes between cities. The size of the node is proportional to the degree. (*b*) Distribution of network degree. The overall feature is that the greater the degree of nodes, the smaller the frequency. (*c*) The median urban population with different degrees and the fluctuation range of 25–75%.

The network consisted of 30 nodes. The larger the degree is, the larger the nodes are. As shown in figure 2, City 5 had the largest degree—16. The average degree of the network was 5.4; the degree of distribution is presented in figure 2*b*. Moreover, the city population distribution for different degrees is shown in figure 2*c*. Considering the computing power and time cost, the city population was set to be equivalent to the size of a small town.

### 3.2. Population mobility

**Figure 3.**
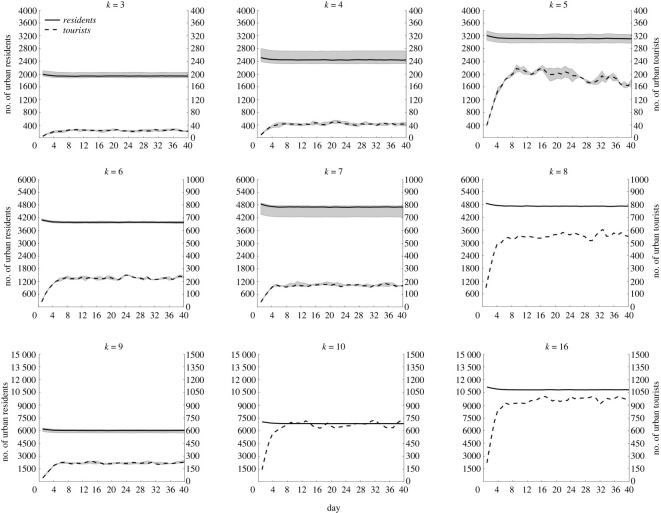
Dynamic changes of city population with different degrees when the population outflow rate is 0.01. The solid line represents the median number of local citizens, measured by the left coordinate axis. The dotted line represents the median number of foreign tourists, measured by the right coordinate axis. The shadow represents the fluctuation range of 25–75%. The graph with *k* = 8, 10 and 16 has no fluctuation range because only one city with these degrees is present.

The number of local citizens in the city decreased slightly in the early stages and remained at a constant level over time, as shown in figure 3. The number of foreign tourists also remained at a certain level after a sharp increase in the early stages and fluctuated in some cities.

### 3.3. Epidemic transmission

#### 3.3.1. Transmission chain

**Figure 4.**
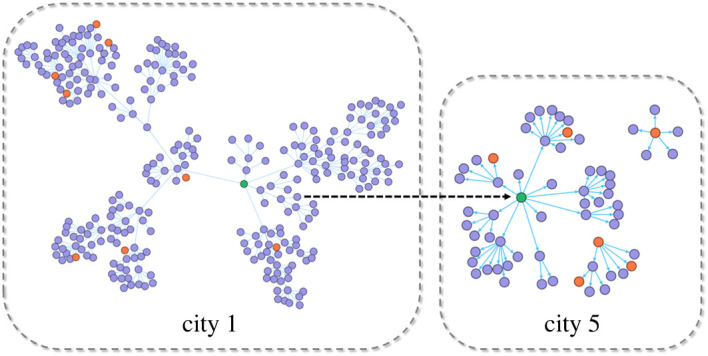
Dynamic process of epidemic spreading from City 1 to City 5. The figure shows the epidemic transmission in City 1 and City 5 from day 0 to day 20 for *R*_0_ = 9 and a population outflow rate of 0.01. The circle, oriented arrow, green circle, purple circle and red circle indicate the infected person, transmission relationship, first case in the city, local infected person, and foreign infected person in the transmission chain, respectively.

The first case emerged in City 1 (figure 4); hence, the epidemic first spread in City 1. Because the local population was larger than the foreign population, most of the infected people were local citizens. However, the epidemic gradually spread to other cities with the population flow. An infected traveller from City 1 entered City 5, spreading the epidemic to the city. In addition, the arrival of infected persons from other cities caused a new epidemic spread in the city over time.

#### 3.3.2. Time distribution of infected persons, patients and inpatients

**Figure 5.**
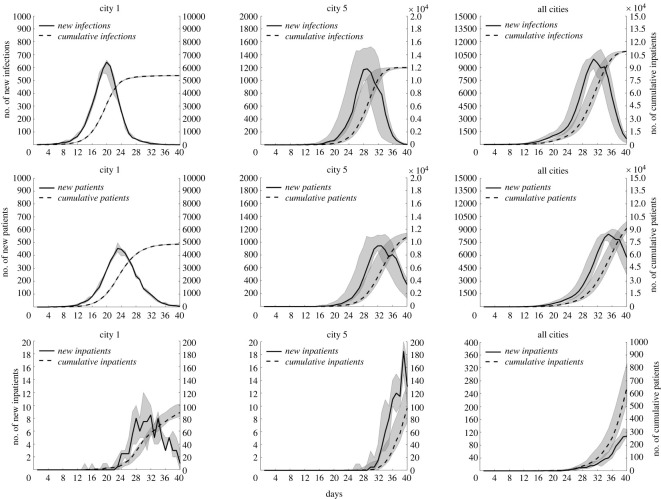
Time distribution characteristics of the epidemic transmission in different cities when *R*_0_ = 9 and a population outflow rate of 0.01. The first to third row represents the time distribution of infected persons, patients and inpatients, respectively. The solid line represents the median number of newly increased persons, measured by the left coordinate axis. The dotted line represents the median of the cumulative number of persons, measured by the right coordinate axis. The shadow represents the fluctuation range of 25–75%.

The epidemic transmission process in City 1, City 5 and the combination of all cities is shown in figure 5. In City 1, the peak time of newly infected persons and new patients was on days 20 and 23, with a median of 641 and 455, respectively. The accumulative number of infected and onset persons on day 40 was 5371 and 4880, respectively. In City 5, the peak time of newly infected persons and new patients was on days 29 and 33, with a median of 1178.5 and 942.5, respectively. The accumulative number of infected and onset persons on day 40 was 11 990 and 10 795, respectively. In all cities, the peak time of newly infected persons and new patients was on days 31 and 35, with a median of 9964 and 8472, respectively. The cumulative number of infected and onset persons on day 40 was 108 640 (with an infection rate of 99.4%) and 91 685 (with an attack rate of 83.9%), respectively. The number of new inpatients fluctuated significantly. The cumulative median number of inpatients in City 1, City 5 and all cities on the 40th day reached 89, 97 and 651, respectively. Note that the infected persons, patients and inpatients in cities included local citizens and foreign tourists.

#### 3.3.3. Effects of different *R*_0_ and population outflow rates on the change in the number of infected persons

**Figure 6.**
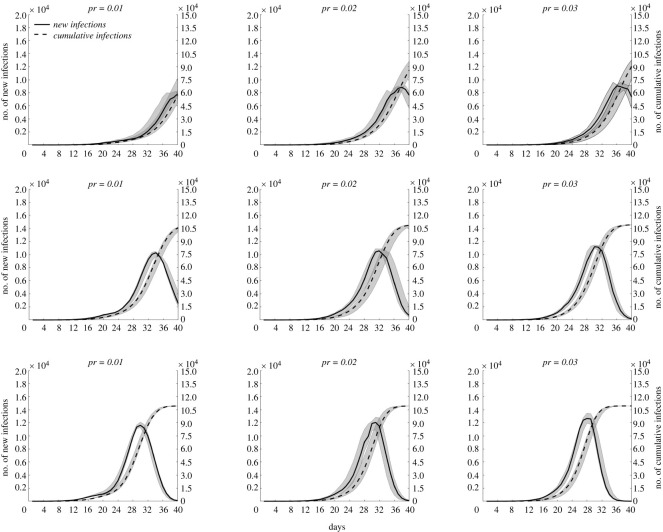
Time distribution characteristics of infected persons with different values of *R*_0_ and population outflow rate (*pr*). The first to third row represents the time distribution of infected persons in the entire network when *R*_0_ = 6, 8 and 10, respectively. The solid line represents the newly infected persons, measured by the left coordinate axis. The dotted line indicates the cumulative infected persons, measured by the right coordinate axis.

As shown in figure 6, at constant *R*_0_ and increasing population outflow rate, a faster increase in the number of newly infected persons in the entire network, earlier peak appearance and larger median at the peak were observed. At constant population outflow rate and increasing *R*_0_*,* similar changes in the number of newly infected persons were observed. This result suggested that *R*_0_ and population outflow rate were positively correlated with the growth rate of infected persons.

#### 3.3.4. Increase trend in the number of infected persons in city networks

**Figure 7.**
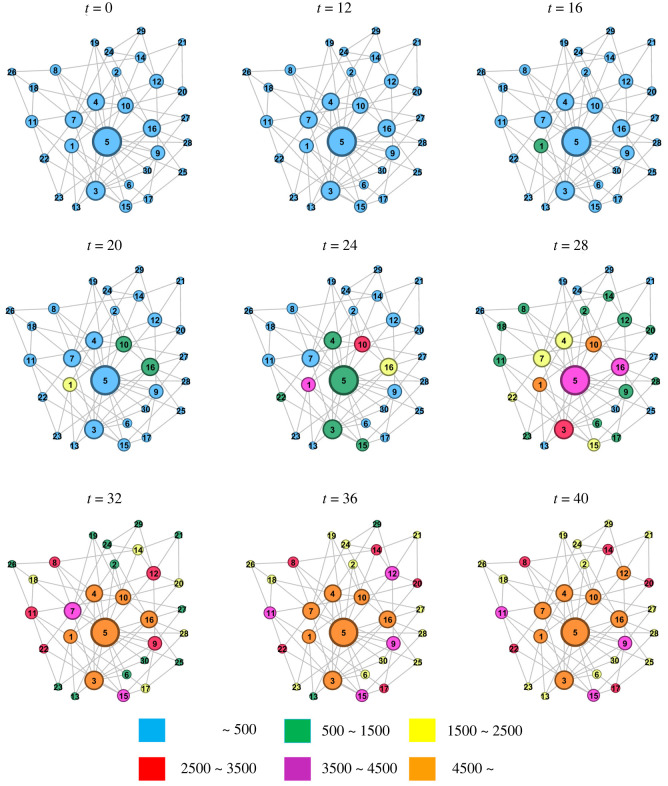
Time distribution of the epidemic spreading in the city network for *R*_0_ = 9 and a population outflow rate of 0.03. The first case emerged in City 1, and the cumulative number of infected persons in different cities at different time points is represented by different colours.

As shown in figure 7, the epidemic spread mainly in City 1 before day 16, with a slow increase in the number of infected persons. Moreover, infected people emerged in other cities and increased rapidly with population flow. Note that the greater the degree of the city, the faster the increase in the number of infected persons. The number of infected persons in all cities reached the theoretical upper limit by day 40.

### 3.4. Sensitivity analyses

**Figure 8.**
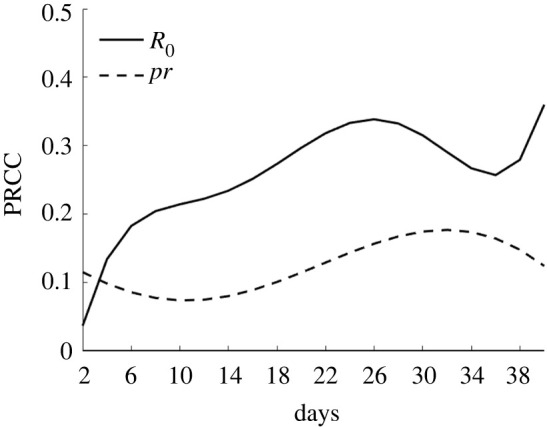
Sensitivity analysis of continuous time.

In this study, sensitivity analyses were conducted with the model based on two parameters (*R*_0_ and population outflow rate) and a continuous time series for the entire network's cumulative number of infected persons. We considered *N* = 50 samples from a uniform distribution for each parameter range. PRCCs near one indicates that the parameter strongly affects the output. By contrast, a value closer to zero indicates that the output result is less affected by the parameter (figure 8). The results reflected that *R*_0_ is positively correlated with the cumulative number of infected persons, which was consistent with the objective facts. The impact of the population outflow rate on the number of infected persons was smaller than that of *R*_0_. This was because the role of the population outflow rate is to spread the epidemic among cities. Even if the population outflow rate is substantially low, the epidemic will spread in cities, which has a relatively limited restrictive effect on the increase in infected persons in the entire network.

## 4. Discussion

### 4.1. Features of the research

Compared with previously published research, the proposed model has the following characteristics: first, compared with several previous studies that considered individuals as nodes and the epidemic transmission through contact as adjacent nodes, this model clearly restored the epidemic transmission process through population mobility among city agglomerations, which was more in line with the objective reality. Second, the population mobility and epidemic transmission was simulated entirely by program design using the individual as a research unit. This approach could microscopically simulate individual behaviour and macroscopically show the overall trends compared with dynamic models based on differential equations. Third, the impact of several epidemic prevention measures, such as blocking urban traffic, performing nucleic acid detection and monitoring foreign tourists using electronic travel records, on the epidemic transmission trend could be discussed by developing an in-depth model based on this research.

### 4.2. Population mobility

In practice, the probability of large cities being selected as travel destinations is higher than that of small cities; thus, the probability of a city selection as a travel destination was considered proportional to the degree of nodes. The simulation results showed that the population mobility did not cause a sustained change in the city population number; however, it remained at a certain level, consistent with the actual situation where the city population number was stable. Figure 3 shows that the local population is relatively stable because the outflow and inflow of the local population are balanced. By contrast, the number of foreign tourists showed significant fluctuations because of the strong randomness of foreign tourists' choice of travel destinations. Moreover, the number of local citizens in the city was positively correlated with the number of tourists, with the exception of *k* = 7. This is because when the program was designed initially, each traveller randomly selected the degree; subsequently, the traveller selected the city with the selected degree when selecting his or her destination. Three cities with a degree of 7 were present. Therefore, the number of tourists selecting one of the cities could be reduced.

### 4.3. Transmission of epidemic

The emergence of the epidemic in all cities was due to the imported cases, except in the city of its origin. Moreover, the higher the population mobility, the higher the number of imported cases. Figure 5 shows that the peak time of newly infected persons in City 1, City 5 and all cities is postponed. This is because the first case emerged later in City 5 than in City 1. Further, all cities represented the overall population of the city agglomeration. Because most cities had a small degree, the peak time of city agglomerations occurred at the time when the epidemic input was later. The number of infected persons and patients in City 1 fluctuated slightly, indicating that the transmission trend of the epidemic was more specific in the original place. Figure 6 shows that an increase in *R*_0_ and population outflow rate promotes epidemic development in city agglomerations. In particular, the higher *R*_0_, the faster the epidemic spread. By contrast, the larger the population outflow rate, the greater the number of floating population, thereby promoting the spread of the epidemic to more cities. Figure 7 shows that as the traffic connections with other cities increases, the floating population in the city increases, the risk of epidemic spread is high and the epidemic spreads rapidly.

### 4.4. Limitations of the model

The limitations of the model were mainly reflected in the following four aspects. First, because a virtual model was established for research convenience, various assumptions were comparatively performed. For example, the setting of parameters, such as population outflow rate, travel planning and travel time of tourists, was simpler than the diversified choices of tourists in the real world. Second, the transmission route of the epidemic is considered the most typical situation of human-to-human transmission; the model excluded the situation in which SARS-CoV-2 could be spread by touching the surface of objects. Third, vaccination, nucleic acid screening, health monitoring, traffic blockade and other epidemic prevention measures were not considered. These measures should be further studied based on the proposed model. Finally, the computational burden of the proposed model is high. If the epidemic transmission is simulated on a large city network or even nationwide, a desktop computer cannot be used to perform the simulations.

## 5. Conclusion

We developed a dynamic model of infectious diseases to discuss the relationship between population mobility and epidemic transmission based on a scale-free network. Moreover, we deeply analysed the mechanism of epidemic transmission in the network. The results showed that *R*_0_ and population outflow rate were positively correlated with the spread speed of the epidemic. Compared with small and medium-sized cities, central cities were more prone to the input and spread of the epidemic. The results suggested that to control the epidemic effectively, the population flow between cities should be reduced. Particularly, the central cities should restrict the migrant population strictly and perform nucleic acid screening and health monitoring on time.

## Data Availability

All parameters analysed during this study are presented in table 1. Program codes of the model are supplied in the electronic supplementary material [20] that can be consulted in Zenodo (https://doi.org/10.5281/zenodo.6814620) [21].
